# Biochemical Verification of Tobacco-Use as an Inclusion Criterion in Smoking Cessation Trials- Lessons From the Cessation of Smoking Trial in the Emergency Department

**DOI:** 10.1177/1179173X231193898

**Published:** 2023-08-14

**Authors:** Ian Pope, Chandhini Suresh, Emma Ward, Pippa Belderson, Caitlin Notley

**Affiliations:** 1 Norwich Medical School, 6106 University of East Anglia, Norwich, UK; 2 12213 Leicester Medical School, Leicester, UK

**Keywords:** smoking cessation, trial methodology, biochemical verification

## Abstract

**INTRODUCTION:**

Biochemical verification of smoking status prior to recruitment into smoking cessation trials is widely used to confirm smoking status, most commonly using exhaled carbon monoxide (CO). There is variation in the level of CO used as a biochemical inclusion criterion, and thus the possibility for people reporting to be current smokers to be incorrectly excluded from trials.

**METHODS:**

As part of the Cessation of Smoking Trial in the Emergency Department, people attending the Emergency Department (ED) who reported being current daily smokers underwent CO testing to confirm eligibility. Elective semi-structured interviews were undertaken with the researchers who recruited participants. As part of the interviews, researchers were asked their views and experiences with CO testing.

**RESULTS:**

Of the 1320 participants who reported being current daily smokers and underwent CO testing, 300 (22.7%) blew a CO reading of 7 ppm or less and were excluded from taking part. Possible explanations offered by researchers for participants blowing low CO readings were (1) long wait times in the ED, therefore a long period having elapsed since people had last smoked and (2) patients having reduced smoking for the period before the ED attendance due to ill health.

**CONCLUSIONS:**

Biochemical verification has the potential to improve internal validity of smoking cessation for inclusion in trials, but at the cost of reduced generalisability through exclusion of participants who would receive the intervention if it were implemented in practice. We would recommend researchers carefully consider whether it is appropriate and necessary to include biochemical verification as an inclusion criterion.

## Introduction

Studies which aim to test the effectiveness of interventions to help people quit smoking are essential in the battle against tobacco related harm. For the results to be valid it is important that only current smokers are recruited, otherwise the true effectiveness of the intervention will not be demonstrated.

The Society for Research on Nicotine and Tobacco (SRNT) Treatment Research Network recommendations on biochemical verification of tobacco use and abstinence state that researchers must decide whether to include biochemical verification of smoking status as an inclusion criterion for studies. The authors state that biochemical verification may be particularly important for trials that involve contingency management or trials involving switching to alternative nicotine products.^1^ This is based on the assumption that people who do not smoke may attempt to join a trial in order to gain a benefit, e.g. receive an incentive. However there is no conclusive evidence of such “gaming” happening in reality.^2^ This is in comparison to the use of biochemical verification at follow-up to confirm cessation where it is widely used and accepted as a ‘gold standard’ outcome.^3^

Of the 78 studies included in the September 2022 Cochrane E-Cigarette systematic review^4^ 17 used biochemical verification at baseline, 57 did not, and 4 did not report it. Of those that used biochemical verification, one used cotinine and the remainder used exhaled carbon monoxide (CO) with thresholds of 5 ppm (3 studies), 6 ppm (3 studies), 8 ppm (1 study), 9 ppm (2 studies) 10 ppm (7 studies) and 15 ppm (1 study). Two studies reported the number of participants excluded as a result of a low CO reading.^5,6^ The Russell standard sought to standardise the measurement and reporting of smoking status in trials with a CO reading of 10 ppm or more signifying smoking.^3^

Carbon monoxide typically has a half-life of around 4 h, meaning someone with a level of 12 ppm would be expected to have a level of 6 ppm 4 h later if they did not smoke during that period.^7^ However, this half-life is influenced by exercise, pregnancy and time of day.^7,8^

Different types of CO monitors have been shown to provide different results with one study of 78 people who smoked finding the Bedfont monitor giving mean CO readings 3.8 ppm higher than the Vitalograph monitor, although the difference was smaller (1.7 ppm) among those who reported abstaining in the prior 12-24 h compared to those who smoked regularly.^9^

Studies that have calculated the sensitivity (i.e. ability to avoid false negatives) of CO verification for smoking status have found sensitivities of between 57.6% and 90% depending on the population and cut off used.^7,10-12^

The lung health study found that at 1 year follow-up 6% of participants in both the intervention and control group who reported current smoking had a CO reading of less than 10 ppm.^13^ It is worth noting that participants in the lung health study were ‘heavy smokers’ with an average of 32.7 cigarettes reportedly smoked per day by men and 28.9 by women at baseline.^14^

## Methods

The Cessation of Smoking Trial in the Emergency Department (COSTED) is a multi-centre randomised controlled trial of a smoking cessation intervention for people attending the Emergency Department.^15^ COSTED used an exhaled CO level of ≥8 ppm as an inclusion criterion. People in Emergency Departments (EDs) were screened by asking if they were currently smoking tobacco. At this point participants did not know why they were being asked. Therefore it seems unlikely that they would report being a person who smokes if they were not, particularly given the stigma around smoking that still exists.^16^ Those who reported smoking were screened for inclusion and exclusion criteria including being asked if they were a “daily tobacco smoker, smoking at least one cigarette (or equivalent) per day”. Those who met inclusion criteria were consented and then underwent CO verification using a Bedfont® piCO Smokerlyzer administered by a trained researcher. Those who blew a reading of ≤8 ppm were excluded from taking part in the trial.

Eleven semi-structured interviews were undertaken with the researchers who were recruiting participants across all 6 participating Emergency Departments as part of the process evaluation. Interviews, conducted via video or voice calls, were audio recorded and transcribed verbatim.

## Results

### Quantitative

Of the 1320 participants who reported being current daily smokers and underwent CO testing 300 (22.7%) blew a CO reading of 7 ppm or less and were excluded from taking part. A further 477 (36.1%) had readings between 8 and 15 ppm. The number of participants who blew each of the CO readings is shown in Table 1.

**Table 1.** Frequency of CO readings amongst potential COSTED participants.

CO reading (ppm)	Frequency (%)
0	3 (.2%)
1	14 (1.1%)
2	44 (3.3%)
3	57 (4.3%)
4	51 (3.9%)
5	47 (3.6%)
6	57 (4.3%)
7	27 (2.1%)
8	90 (6.8%)
9	72 (5.5%)
10	60 (4.6%)
11	56 (4.2%)
12	53 (4.0%)
13	46 (3.5%)
14	43 (3.3%)
15	57 (4.3%)
16 or more	543 (41.1%)

### Qualitative

Possible explanations offered by a large number of researchers for participants blowing low CO reading were (1) long wait times in the ED therefore a long period having elapsed since people had last smoked and (2) patients having reduced smoking for the period before the ED attendance due to ill health.

Other speculative explanations the advisors suggested were (1) younger, fitter people tending to blow lower CO readings; (2) the way people smoke potentially contributing, e.g. not inhaling deeply; (3) possible measurement error and (4) people with chronic lung condition and facial palsies being unable to exhale fully to complete the CO test.

Advisors reported the consequences of excluding participants based on low CO readings as being (1) people being upset that they were not being given a chance to participate, (2) false reassurance to smokers who blew a low reading that they were not at risk and (3) exclusion of smokers with chronic lung conditions from the trial.

## Discussion

Using biochemical verification as an inclusion criterion for smoking cessation trials has the advantage of preventing people who do not smoke from joining trials, thus potentially improving internal validity. The disadvantages of this approach are (1) the potential to exclude people reporting current smoking from taking part, therefore limiting the generalisability of the results given any intervention is likely to be offered to all self-reported smokers if implemented in practice; (2) the cost and time taken to complete the biochemical verification at baseline; (3) it being based on the assumption that participants would lie about their smoking status at baseline, which is not supported by evidence, and may suggest a power relationship between participant and researcher within the trial setting which may not be desirable and; (4) the ethical issue of denying those who report current smoking the opportunity of taking part in a trial.

In the COSTED trial we excluded a large number of participants who potentially may have benefitted from the intervention. The mechanism for this is likely to be the long wait time in the ED when people were not smoking. Given the adverse consequences on generalisability, the limitations of CO readings in certain populations, the uncertainty around the optimal CO level to use and the lack of rationale for using biochemical verification as an inclusion criterion, in hindsight we would not have used biochemical verification at baseline. A limitation of the data presented is that there was no alternative way to confirm smoking status, e.g. cotinine, therefore we must rely on self-report. An alternative approach would be to use cotinine which has a longer half-life, however this would not discriminate between people who smoke and those using e-cigarettes or nicotine replacement therapy.^1^

We recommend only including biochemical verification of smoking status as an inclusion criterion for clinical trials where there is clear rationale for its use. For most “real world” trials we believe it is not necessary and limits generalisability of the results and transferability to routine care. Where biochemical verification is used we recommend adopting the Russell standard and reporting of the number of participants excluded as a result of the inability to confirm smoking status.
